# WORK FORCE COMPETENCIES AND TRAINING FOR SELF-DIRECTED SERVICE PROGRAMS

**DOI:** 10.1093/geroni/igz038.861

**Published:** 2019-11-08

**Authors:** Mark Sciegaj, Nancy Hooyeman

**Affiliations:** 1 Penn State University, University Park, Pennsylvania, United States; 2 University of Washington, Seattle, Washington, United States

## Abstract

In 2017 over one million individuals of all ages were enrolled in approximately 260 self-directed long-term services and support programs nationwide. Research conducted by the National Resource Center for Participant-Directed Care (NRCPDS) and the Council for Social Work Education identified training gaps among current aging and disability network professionals and within social work education. Believing that both self-directing individuals and their family caregivers would benefit from a workforce that has the knowledge and skills to implement the principles of self-directed care, NRCPDS and CSWE working with national professional organizations and government agencies have identified workforce competency domains and developed a number of training resources that can be used in both academic and professionals settings. This presentation will review the work of NRCPDS and CSWE in workforce competencies, training resources, and recommendations for self-directed services training.

